# Inappropriate long-term use of antipsychotic drugs is common among people with dementia living in specialized care units

**DOI:** 10.1186/2050-6511-14-10

**Published:** 2013-02-08

**Authors:** Maria Gustafsson, Stig Karlsson, Hugo Lövheim

**Affiliations:** 1 Maria Gustafsson, Department of Pharmacology and Clinical Neuroscience, Umeå University, 901 85, Umeå, Sweden; 2 Stig Karlsson, Department of Nursing, Umeå University, Umeå, Sweden; 3 Hugo Lövheim, Department of Community Medicine and Rehabilitation, Geriatric Medicine, Umeå University, Umeå, Sweden

**Keywords:** Antipsychotic prescribing, Dementia, BPSD, Inappropriate prescribing, Aggression, Passiveness

## Abstract

**Background:**

Antipsychotic drugs are widely used for the treatment of Behavioral and Psychological Symptoms of Dementia (BPSD), despite their limited efficacy and concerns about safety. The aim of this study was to describe antipsychotic drug therapy among people with dementia living in specialized care units in northern Sweden.

**Methods:**

This study was conducted in 40 specialized care units in northern Sweden, with a total study population of 344 people with dementia. The study population was described in regard to antipsychotic drug use, ADL function, cognitive function and BPSD, using the Multi-Dimensional Dementia Assessment Scale (MDDAS). These data were collected at baseline and six months later. Detailed data about antipsychotic prescribing were collected from prescription records.

**Results:**

This study showed that 132 persons (38%) in the study population used antipsychotic drugs at the start of the study. Of these, 52/132 (39%) had prescriptions that followed national guidelines with regard to dose and substance.

After six months, there were 111 of 132 persons left because of deaths and dropouts. Of these 111 people, 80 (72%) were still being treated with antipsychotics, 63/111 (57%) with the same dose. People who exhibited aggressive behavior (OR: 1.980, CI: 1.515-2.588), or passiveness (OR: 1.548, CI: 1.150-2.083), or had mild cognitive impairment (OR: 2.284 CI: 1.046-4.988), were at increased risk of being prescribed antipsychotics.

**Conclusion:**

The prevalence of antipsychotic drug use among people with dementia living in specialized care units was high and inappropriate long-term use of antipsychotic drugs was common.

## Background

Dementia is a disorder that causes permanent and progressive impairment of cognitive functions such as memory and other cognitive abilities [1]. Behavioral and Psychological Symptoms of Dementia (BPSD) is the term applied to the various problems that complicate dementia and prevalence is high. It is estimated that up to 90% of patients with Alzheimer’s disease may present at least one BPSD during the course of the disease [2]. BPSD include behaviors such as aggression, screaming, restlessness, and also symptoms such as anxiety, hallucinations and depressive mood [3].

Antipsychotic drugs are widely used for the treatment of certain BPSDs; one study showed that 40% of elderly people with cognitive impairment living in group dwellings took antipsychotic drugs [4]. However, antipsychotic drugs have demonstrated only limited efficacy in the treatment of BPSD. A systematic review found that first generation antipsychotics had little efficacy at best, and that the benefits may not outweigh the risk of side effects [5]. The same review showed that olanzapine and risperidone had a modest, statistically significant efficacy with minimal adverse effects at lower doses [5]. However, in another study, adverse effects offset the clinical benefit of second generation antipsychotics for the treatment of BPSD [6].

Older people tend to experience side-effects more frequently and with greater severity than younger people, and antipsychotic drugs have a number of side-effects. First generation antipsychotics are associated with a high prevalence of extrapyramidal side-effects (EPS) and tardive dyskinesia due to their high affinity for D_2_-receptors [7]. Second generation antipsychotics have a different receptor-binding profile. Since they interact with both D_2_ and 5HT_2_-receptors, they cause EPS to a lesser extent [8]. The use of antipsychotic drugs appears to be associated with accelerated cognitive decline in people with Alzheimer’s disease; treatment with haloperidol over 6–8 weeks was associated with a decline in cognition measured using the Mini-Mental State Examination (MMSE) [9]. However, one study showed that the MMSE score did not worsen after treatment with risperidone among people with dementia [10], which might be due to the lack of anticholinergic activity of risperidone [11]. All antipsychotics seem to cause metabolic side-effects. However, it appears that second generation antipsychotics are worse in this regard, even if there is variation among substances in this group [12].

Second generation antipsychotics have also been associated with an increased risk of cerebrovascular events and increased mortality, and first generation antipsychotics seem to carry the same risk [13,14].

Support for the long-term use of antipsychotics in this patient population is limited. There is some evidence favoring short-term use, [1,15] even if it is considered an option of last resort. Even short-term use of antipsychotics increases the risk of serious adverse events [16]. According to the Swedish Medical Products Agency, the dose should initially be low and then titrated upwards and treatment should be time-limited and regularly reviewed [1]. Only two recent studies have described long-term antipsychotic treatment among people with dementia [17,18] and both found relatively high rates of long-term treatment. There is still a need for detailed study of the long-term use of antipsychotic in this population. The aim of this retrospective study was to describe the prevalence, associated factors and long-term use of antipsychotic drugs among people with dementia living in specialized care units.

## Methods

### Subjects and settings

The study population for this project has previously been used in a research study concerning use of physical restraint [19]. This was an intervention study conducted in 2005-2006, which included 40 specialized care units (353 people) in nine communities in northern Sweden. These units are designed to provide care for six to eight persons with dementia in homelike environments. All specialized care units in these communities were inventoried, i.e. 99 units were contacted - and units with the highest prevalence of physical restraint use (≥20%) were selected. In the present study, 9 people were excluded due to incomplete data. The study was approved by the Regional Ethical Review Board in Umeå (registration number 02-105).

### Procedures

Data were collected by means of a questionnaire completed by care unit staff, the Multi-Dimensional Dementia Assessment Scale (MDDAS) [20]. The MDDAS has good intra- and inter-rater reliability [20]. This instrument includes assessment of the level of functioning in activities of daily living (ADL), cognition and behavioral and psychological symptoms. ADL function score ranges from 4-24, where a higher score indicates greater ADL independence. This score is based on the person’s ability to cope with hygiene, dressing, eating and bladder and bowel control. Cognitive function was measured using an assessment scale developed by Gottfries and Gottfries [21]. This scale ranges from 0-27 points and a score of less than 24 is considered to indicate cognitive impairment, correlating with a sensitivity of 90% and a specificity of 91% to the usual cut-off point of the MMSE (24/30) [21,22]. The scale is further subdivided into three groups - mild cognitive impairment (16-23), moderate cognitive impairment (8-15) and severe cognitive impairment (0-7). The MDDAS contains 25 behavioral items and 14 psychological symptom items. Each item is rated on a three-point scale indicating that the symptom was present at least once a day, once a week, or never during the observation period of one week. These variables are dichotomized between at least once a week and less than once a week in the present study.

All persons’ prescription records were collected at the start of the study and six months later. The majority of people dispensing service where the used an automated multidose dispensing service where the persons’ drugs are dispensed in one dose unit bag for each dose occasion.

The prescription records were searched to identify those people from the study population treated with antipsychotic drugs. All people were listed by age, sex, and treatment with antidepressants (N06A), anxiolytics, hypnotics and sedatives (N05B&C), anti-dementia drugs (N06D), and antipsychotics (N05A). The WHO ATC (Anatomical Therapeutic Chemical Index) classification system was used.

Information about dose and type of antipsychotic drugs was collected, and also indication for treatment when this was reported in the prescription records. These indications are written by the prescribing physicians to describe for the patient and nursing staff why the patient is prescribed the drug. Also, the pharmacy uses the indication to check the appropriateness of drug choice and dose.

Lithium (N05AN01) was not included since it differs from antipsychotics regarding both mechanism of action and use. Pro re nata (PRN) drugs were also not included, as information was lacking on the actual use of these drugs, and furthermore very few people used PRN antipsychotics.

To be able to compare antipsychotic doses between baseline and follow-up, we calculated all antipsychotics in haloperidol equivalents. Recommendations concerning antipsychotic use vary slightly in different guidelines [1,15]. Since this study was conducted in Sweden, we used the guidelines from the Swedish Medical Products Agency to evaluate the appropriate use of antipsychotics [1]. These guidelines state that risperidone is the only antipsychotic drug that is labeled for use in combating BPSD in Sweden, and the recommended dose is ≤1.5 mg daily. According to the same guidelines, the indications that justify the prescribing of antipsychotic drugs in people with BPSD are psychotic symptoms and aggressive behavior that causes suffering or potential danger for the person or others.

### Statistics and calculations

People who did and did not take antipsychotics were compared using the Pearson chi-square test and t-test for dichotomous and continuous variables respectively. SPSS 18 for MacOS X was used for data handling and statistical calculations. A p-value of < 0.05 was considered statistically significant. A multiple logistic regression model was constructed to find factors independently associated with antipsychotic drug use. The behavioral and the psychological symptom items of the MDDAS were grouped and weighted (in each group every symptom was multiplied with the calculated factor loading and then added with next symptom) according to a factor analysis previously described by Lövheim et al [23]. The factors were then normalized and included in a logistic regression model which also included age, sex and level of cognitive impairment. As many of the behavioral and psychological symptoms correlated strongly, the behaviors and symptoms were tested in the regression model in a stepwise procedure, where the behavior that had the strongest bivariate correlation (aggressive behavior) was included first, and all other behaviors and symptoms were included subsequently one by one to see if any of them contributed independently. These behaviors and factors, apart from aggressive behavior, were: wandering behavior, restless behavior, verbally disruptive/ attention-seeking behavior, passiveness, hallucinatory symptoms, depressive symptoms, disoriented symptoms and regressive/inappropriate behavior. Ultimately, all significant behaviors and symptoms were included in a final model.

Survival among people who were treated with antipsychotic drugs at the start of the study was compared with those who were not treated with antipsychotics, using a Cox regression, also including age, sex and level of cognitive impairment.

## Results

The study population comprised 344 people with dementia whose characteristics are presented in Table 1. One hundred and thirty-two (38%) of these people used antipsychotic drugs at the start of the study; 118 people were prescribed one antipsychotic drug, 13 took two, and 1 person had three antipsychotic drugs prescribed concomitantly. Ninety prescriptions were for second generation antipsychotics, and 57 for first generation antipsychotics. There were no associations between antipsychotic drug use and antidepressant drug use, or between antipsychotic drug use and anti-dementia drug use. However, there was an association between antipsychotic drug use and anxiolytic, hypnotic and sedative drug use, as shown in Table 1. An association was found between antipsychotic drug prescribing and age, but no difference between men and women.

**Table 1:** Characteristics of study population and comparison between people with and without antipsychotics

	***With AP***	***Without AP***	***Total***	***p-value***
Cases, n (%)	132 (38.4)	212 (61.6)	344	
Women, n (%)	92 (69.7)	153 (72.2)	245 (71.2)	0.62
Mean age ± SD	80.9 ± 8.6	82.9 ± 7.2	82.1 ± 7.8	0.02
ADL score (4-24) mean ± SD	11.9 ± 5.2	12.2 ± 5.4	12.1 ± 5.3	0.63
Cognitive score (0-27) mean ± SD	10.6 ± 7.4	10.0 ± 7.2	10.2 ± 7.3	0.47
Antidepressant (N06A) use, n (%)	73 (55.3)	107 (50.5)	180 (52.3)	0.38
Anxiolytics, hypnotics and sedatives (N05B&C) use, n (%)	72 (54.5)	84 (39.6)	156 (45.3)	0.007
Anxiolytics (N05B) use, n (%)	28 (21.2)	18 (8.5)	46 (13.4)	0.001
Hypnotics and sedatives (N05C) use, n (%)	63 (47.7)	76 (35.8)	139 (40.4)	0.029
Anti-dementia drugs (N06D) use, n (%)	26 (19.7)	45 (21.2)	71 (20.6)	0.733

SD= Standard Deviation, ADL= activities of daily living, AP= Antipsychotic drug.

The multiple logistic regression analysis showed that those who exhibited an aggressive behavior, or passiveness, were younger or had mild cognitive impairment as compared to severe cognitive impairment, were at increased risk of being prescribed an antipsychotic drug (Table 2).

**Table 2:** Multiple logistic regression of antipsychotic drug use

	***Odds***	***95%***	***p-value***
	***Ratio***	***confidence***	
		***interval***	
Male sex	0.969	0.546-1.719	0.913
Higher age	0.958	0.924-0.993	0.018
Moderate cognitive impairment^a^	1.802	0.973-3.338	0.061
Mild cognitive impairment^a^	2.284	1.046-4.988	0.038
Aggressive behavior	1.980	1.515-2.588	<0.001
Passiveness	1.548	1.150-2.083	0.004

Model Cox and Snell R^2^: 0.129, concordance between observed and predicted value: 67.6%. ^a^ Cognitive score ranges from 0-27 points and a score of less than 24 is considered to indicate cognitive impairment. The scale is subdivided into three groups, 0-7 (severe cognitive impairment), 8-15 (moderate cognitive impairment) and 16-23 (mild cognitive impairment). Severe cognitive impairment is reference category.

**Figure 1:**
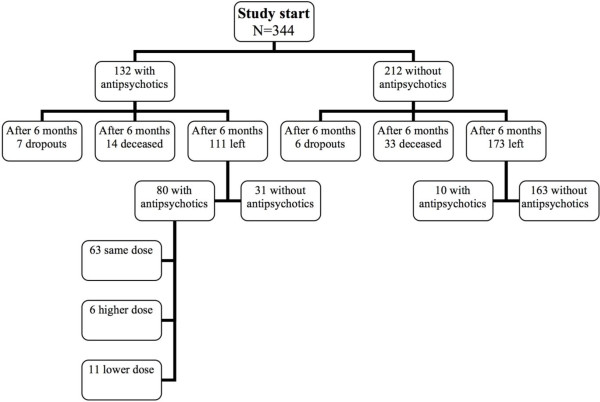
Flow chart of participants from baseline to 6-month follow-up.

After six months, 111 people remained to be evaluated of those who were treated with antipsychotics at baseline (7 dropouts, 14 deceased), as presented in Figure 1. Of these 111 people, 80 (72%) were still being treated with antipsychotics, 63 of these with the same dose. Seventy-eight people of 80 were taking the same antipsychotics as before. Of those who were not treated with antipsychotics at baseline, 10 people were receiving antipsychotics at the 6- month follow-up. After six months, 31 persons had ended their treatment with antipsychotics. The mean age of this group was 83.1, compared to 80.0 among those who still were using antipsychotics after six months (80 persons). However, this difference was not significant. No significant differences were seen concerning ADL, cognitive score or sex.

The mortality analysis showed no difference in mortality between those who received antipsychotics at the start of the study and those who did not (OR 0.69, CI 0.36-1.32, p-value 0.26).

At the start of the study, 132 people were prescribed antipsychotic drugs; 62 of these received risperidone, the only antipsychotic drug that is labeled for use in BPSD in Sweden, as shown in Table 3. Of these 62, 52 received the recommended dose, i.e. ≤1.5 mg daily. Hence, 52/132 (39%) received both recommended antipsychotic drug and the recommended dose, assuming that antipsychotics were used solely for the treatment of BPSD.

**Table 3:** Characteristics of antipsychotic drugs at the start of the study

***Drug***	***n***	***Percent (%) of prescribings***	***Dose, mean ± SD***	***Median Dose (mg)***	***Range (mg)***	***Mean Dose (mg) Haloperidol equivalents***	***Halo-peridol***
							***1 mg equivalent doses***^***a***^
***First generation AP***							
Haloperidol	25	17.0	2.1±3.1	1.0	0.5-16	2.1	1
Levomepromazine	12	8.1	19.6±15.6	15.0	5-50	0.4	50
Zuclopenthixol	6	4.1	3.7±2.0	3.0	2-6	0.7	5
Dixyrazine	5	3.4	32.0±38.3	20.0	10-100	1.1	30
Melperone	4	2.7	33.8±11.1	30.0	25-50	0.8	40
Perphenazine	4	2.7	7.8±5.9	6.0	3-16	1.9	4
Chlorpromazine	1	0.7	100.0			2.0	50
***Second generation AP***							
Risperidone	62	42.2	1.1±1.1	0.8	0.25-8	1.1	1
Olanzapine	18	12.2	6.9±5.1	5.0	3-20	2.3	3
Ziprasidone	6	4.1	60.0±31.0	50.0	40-120	1.5	40
Clozapine	3	2.0	216.7±332.0	25.0	25-600	4.3	50
Quetiapine	1	0.7	50.0			0.3	150

Number of prescriptions=147, number of persons taking AP=132, AP=antipsychotic drugs. ^*a*^[24].

The indications for prescribing antipsychotic drugs to this patient group are listed in Table 4. The most common indication was “treatment of disturbed and restless behavior/sedative”. No indication was listed for 19 prescriptions. After six months, ten people who did not receive antipsychotics at baseline, had been started on antipsychotic drugs. Of these ten, one received risperidone and nine people received other antipsychotics. The dose of risperidone of this one person, however, was higher than the recommended dose. The most common indication was “treatment of disturbed and restless behavior/sedative”, similar to those having antipsychotics at study start.

**Table 4:** Indications for antipsychotic treatment at the start of the study

	
Treatment of disturbed and restless behavior/sedative n (%)	67 (42.9)
Delusions/hallucinations/paranoia n (%)	16 (10.3)
Treatment of mood/irritability/anxiety, n (%)	14 (9.0)
Treatment of aggression, n (%)	13 (8.3)
Treatment of insomnia, n (%)	12 (7.7)
Treatment of psychosis, n (%)	7 (4.5)
Treatment of confusion, n (%)	3 (1.9)
To combat behavioral disorders/BPSD, n (%)	2 (1.3)
Other indications, n (%)	3 (1.9)
No indication, n (%)	19 (12.2)

Indication at baseline. Number of prescriptions=147, number of persons taking antipsychotics=132. The number of indications (156) is higher since there were sometimes more than one indication per prescription.

## Discussion

This study showed that many people with dementia who lived in specialized care units were prescribed antipsychotic drugs for long periods. It seems that in most cases the doses were probably not regularly adjusted; a majority of the people appeared to be on stable doses for six months or possibly longer. There were also few people in our study who had been prescribed the drugs in agreement with current recommendations concerning dosage and drug choice. The study showed that people who exhibited aggressive behavior, or passiveness, or had a higher cognitive score, were at increased risk of being prescribed antipsychotics. Those who received antipsychotics were also significantly younger. We found no difference between men and women concerning antipsychotic drug use.

Furthermore, the use of more than one psychotropic drug seemed to be common, 72/344 had anxiolytics/ hypnotics/sedatives and an antipsychotic drug prescribed simultaneously and 73/344 had antidepressants and an antipsychotic drug prescribed simultaneously. In addition, 14 persons had more than one antipsychotic drug prescribed.

The present results are in accordance with previous studies. One study found that people received psychotropic drugs over at least one year despite uncertainty about symptom improvement and another study showed that most antipsychotic prescriptions remained unchanged over a six-month period [17,18]. In the present study, 63/111 (57%) received exactly the same antipsychotic dose after six months. The high prevalence of long-term use is not in line with current recommendations which emphasize that treatment should be time-limited and regularly reviewed [15]. Selbæk *et al* also demonstrated that most symptoms show an intermittent course which does not support long-term treatment with antipsychotics [18]. O’Connor *et al* discuss the fact that the person’s symptoms are classified as present when in reality they occur only occasionally [17]. These findings stress the importance of reviewing antipsychotic use regularly to ensure that the indication remains. One study also showed that dementia persons’ symptoms remain stable when they are withdrawn from first generation antipsychotics, and another study found that people actually improved when second generation antipsychotics were withdrawn [25,26].

Furthermore, the indications that were given for the prescriptions in our study were not in line with the recommendations. By far the most common indication in this study was “treatment of disturbed and restless behavior/sedative”, and this is not an approved indication, according to the guidelines. Some indications were doubtful and in many cases were missing. However, these results should be interpreted with caution since the indications often overlap and the way of expression might differ between physicians. The choice of antipsychotic drugs among prescribers in this study was somewhat surprising considering that the second generation antipsychotics risperidone and olanzapine seem to have the best evidence-base for effectiveness, compared to placebo for physical aggression, agitation and psychosis [27,28]. Risperidone and haloperidol were the most commonly used antipsychotics in our study, which is to be expected. Haloperidol has little anticholinergic activity and was by many considered the most preferable antipsychotic to people with dementia before the introduction of the second generation antipsychotics. An established treatment tradition might possibly have delayed the switch to second generation drugs and explain why many old people with dementia were still treated with haloperidol in 2006. Haloperidol has some efficacy against behavioral problems in higher doses, but its use is limited by side-effects [1]. Risperidone on the other hand is a well-tolerated alternative among people with dementia in lower doses [29] and is, as stated above, the only antipsychotic drug that is labeled for use in BPSD in Sweden. The proportion of people with antipsychotics treated with second generation drugs will probably continue to increase [30].

A more unexpected finding was the fact that these two drugs did not account for a larger share of the antipsychotic prescriptions. Many older first generation antipsychotics and also some of the newest second generation antipsychotics were used to treat BPSD in this patient group. The reason prescribing physicians deviated from current guidelines is unclear. This study was conducted in 2005 and 2006, i.e. 2-3 years before the guideline was issued. Possibly, the prescription may have changed due to new recommendations, however, we still find it relevant to compare the treatment with what is now considered to be appropriate medication.

However, there are other possibilities for treating BPSD in persons with dementia. Primarily, non-pharmacological approaches are recommended, such as investigation /survey of symptoms, possible causes and triggering moments. It is also important to review current pharmacological treatment and consider discontinuation of drugs with potentially adverse effects on the central nervous system and finally, to optimize the care environment and treatment [1,31]. For example, it has been shown that music, physical exercise and recreation might have some effect considering psychological symptoms in people with dementia [32]. When it comes to pharmacological treatment, memantine, cholinesterase inhibitors and SSRI have shown positive efficacy in various studies [33-39]. Among anti-dementia drugs, memantine appears to reduce specific problems such as agitation and irritability [34]. Concerning cholinesterase inhibitors, one meta-analysis showed that rivastigmine had positive effects on nonpsychotic and psychotic symptoms associated with Alzheimer’s disease [35]. Among antidepressants, citalopram has for example showed significant efficacy against behavioral disturbances in individuals with dementia [36,37], and sertraline has showed efficacy against aggressive behavior [38]. Selective serotonin reuptake inhibitors are also recommended as first line treatment for irritability, agitation and anxiety among people with dementia [1].

The association found between antipsychotics and aggressive behavior, as well as the association between antipsychotic use and lower age, confirms the results of an earlier study [4]. The increased risk of receiving antipsychotic treatment among people with aggressive behavior might be expected since this is one of the approved indications for antipsychotics. We also found an association between use of antipsychotics and a higher cognitive score. It has been shown that the prevalence of the behaviors and symptoms decline in those with severe cognitive impairment, and this might possibly lead to less use of antipsychotics [23]. There was also an association between passiveness and use of antipsychotics in the present study. It has been shown that passiveness increases almost linearly with the severity of cognitive impairment [23]. It can be difficult to know what is cause and what is effect, but the passiveness shown among those who use antipsychotic drugs might also, in some cases, be a side-effect of the antipsychotics.

This study did not show any difference in mortality between those who received antipsychotics at the start of the study and those who did not. Several studies have reported an increased mortality among people prescribed antipsychotics, [13,14] while other studies have not - for example one study that found no association between antipsychotics and cerebrovascular events compared to benzodiazepines [40]. A selection effect, where the healthier persons were possibly prescribed antipsychotics more frequently, might have contributed to our results considering mortality and antipsychotics. We did not know the length of exposure to antipsychotics, only that a person was treated with an antipsychotic drug at the start of the study and this have possibly influenced the results. Also, in this study we lacked information about the prevalence of cerebrovascular diseases and other co-morbidities that might have impacted on mortality.

In this study we have been able to describe in detail long-term use of antipsychotics among people with dementia. The registration of drugs and doses in the present study was of high quality. We can also assume that compliance was high since the vast majority of patients used an automated dose dispensing system.

The study also has some methodological limitations. The selection of specialized care units was not random but based on the prevalence of physical restraint use. It could be that people in these homes have severe problems with BPSD and, therefore, receive long-term treatment to a greater extent. In the physical restraint study [19] there was no difference in antipsychotic use within groups or between groups at baseline and after six months, but we have not been able to compare data with non-selected units since we do not have that information. However, the proportion of those who were on antipsychotic drugs does not appear to differ from those found in other studies [4]. We believe that this does not affect the main results of the study, but it should be borne in mind when interpreting the results.

Data were registered at the start of the study and six months later, but what happened between those times is not known, except for mortality. We do not know the duration of antipsychotic treatment at the time of recruitment into the study, and we also do not know if any attempts of dose reduction or attempts of non-pharmacological treatment of BPSD have been made. Further, we do not know the background or other diseases of the participants, and we lack information about adverse effects of antipsychotics e.g. extrapyramidal effects or falls.

In our study, there could possibly have been reasons other than BPSD for prescribing antipsychotics. Some people might have schizophrenia or other chronic psychotic illnesses where recommendations about dose and substance differ from recommendations among people with dementia. This might, to some extent, explain the use of other antipsychotic drugs or higher doses.

Still, the reason for prescribing antipsychotics is probably related to BPSD in the vast majority of cases, among old people with dementia living in specialized care units.

## Conclusion

The prevalence of antipsychotic drug use among people with dementia living in specialized care units was high and inappropriate long-term use of antipsychotic drugs was common. The prescriptions were often not in agreement with current recommendations.

## Competing interests

The authors declare no conflict of interests.

## Authors’ contributions

SK was responsible for the study concept, design and acquisition of subjects. MG reviewed the data for a second time and HL made the statistical analysis. MG and HL analyzed and interpreted the data and prepared the manuscript. All authors critically revised the manuscript, added their comments and approved the final version.

## Pre-publication history

The pre-publication history for this paper can be accessed here:

http://www.biomedcentral.com/2050-6511/14/10/prepub

## References

[B1] Läkemedelsverket Beteendemässiga och psykiska symtom vid demenssjukdom – BPSD [In English: Drug therapy and treatment for Behavioral and Psychological Symptoms of dementia – BPSD. Information from the Medical Products Agency] Retrieved June 10, 2012 from http://www.lakemedelsverket.se/upload/halso-och-sjukvard/behandlingsrekommendationer/BPSD_bakgrund_webb.pdf

[B2] Liperoti R Pedone C Corsonello A Antipsychotics for the treatment of Behavioral and Psychological Symptoms of Dementia (BPSD) Current Neuropharmacology 2008 6 117 124 10.2174/157015908784533860 19305792 PMC2647149

[B3] Cerejeira J Lagarto L Mukaetova-Ladinska EB Behavioral and psychological symptoms of dementia Frontiers in Neurology 2012 3 73 22586419 10.3389/fneur.2012.00073 PMC3345875

[B4] Lövheim H Sandman PO Kallin K Karlsson S Gustafson Y Relationship between antipsychotic drug use and behavioral and psychological symptoms of dementia in old people with cognitive impairment living in geriatric care Int Psychogeriatr 2006 18 713 26 10.1017/S1041610206003930 16879762

[B5] Sink KM Holden KF Yaffe K Pharmacological treatment of neuropsychiatric symptoms of dementia: a review of the evidence J Am Med Assoc 2005 293 596 608 10.1001/jama.293.5.596 15687315

[B6] Schneider LS Tariot PN Dagerman KS Davis SM Hsiao JK Ismail MS Lebowitz BD Lyketsos CG Ryan JM Stroup TS Sultzer DL Weintraub D Lieberman JA CATIE-AD Study Group Effectiveness of atypical antipsychotic drugs in patients with Alzheimer’s disease N Eng J Med 2006 355 1525 1538 10.1056/NEJMoa061240 17035647

[B7] Neil W Curran S Wattis J Antipsychotic prescribing in older people Age Ageing 2003 32 475 483 10.1093/ageing/afg061 12957993

[B8] Gareri P De Fazio P Stilo MA Ferreri G De Sarro G Conventional and atypical antipsychotics in the elderly: a review Clin Drug Investig 2003 23 287 322 10.2165/00044011-200323050-00001 17535043

[B9] Gareri P Cortoneo A Marchisio U Curcio M De Sarro G Risperidone in the treatment of behavioral disorders in elderly patients with dementia Arch Gerontol Geriatr Suppl 2001 7 173 82 11431062 10.1016/s0167-4943(01)00137-6

[B10] Gareri P De Fazio P De Fazio S Marigliano N Ferreri Ibbadu G De Sarro G Adverse effects of atypical antipsychotics in the elderly: a review Drugs Aging 2006 23 937 56 10.2165/00002512-200623120-00002 17154659

[B11] Devanand DP Sackeim HA Brown RP Mayeux R A pilot study of haloperidol treatment of psychosis and behavioral disturbance in Alzheimer's disease Arch Neurol 1989 46 854 857 10.1001/archneur.1989.00520440036018 2667504

[B12] Melkersson KI Dahl ML Hulting AL Guidelines for prevention and treatment of adverse effects of antipsychotic drugs on glucose-insulin homeostasis and lipid metabolism Psychopharmacology 2004 175 1 6 15221198 10.1007/s00213-004-1922-7

[B13] Schneider LS Dagerman KS Insel P Risk of death with atypical antipsychotic drug treatment for dementia: meta-analysis of randomized placebo-controlled trials J Am Med Assoc 2005 294 1934 1943 10.1001/jama.294.15.1934 16234500

[B14] Gill SS Bronskill SE Normand SL Anderson GM Sykora K Lam K Bell CM Lee PE Fischer HD Herrmann N Gurwitz JH Rochon PA Antipsychotic drug use and mortality in older adults in dementia Ann Intern Med 2007 146 775 786 17548409 10.7326/0003-4819-146-11-200706050-00006

[B15] National Institute for Health and Clinical Excellence (NICE) Dementia - Supporting people with dementia and their careers in health and social care Retrieved June 24, 2012 from: http://www.nice.org.uk/nicemedia/live/10998/30318/30318.pdf

[B16] Rochon PA Normand SL Gomes T Gill SS Anderson GM Melo M Sykora K Lipscombe L Bell CM Gurwitz JH Antipsychotic therapy and short-term serious events in older adults with dementia Arch Intern Med 2008 168 1090 1096 10.1001/archinte.168.10.1090 18504337

[B17] O’Connor DW Griffith J McSweeney K Changes to psychotropic medications in the six month after admission to nursing homes in Melbourne, Australia Int Psychogeriatr 2010 22 1149 53 10.1017/S1041610210000165 20199701

[B18] Selbæk G Kirkevold Ø Engedal K The course of psychiatric and behavioural symptoms and the use of psychotropic medication in patients with dementia in Norwegian nursing homes a 12-month follow-up study Am J Geriatr Psychiatry 2008 16 528 536 10.1097/JGP.0b013e318167ae76 18591573

[B19] Pellfolk TJ Gustafson Y Bucht G Karlsson S Effects of a restraint minimization program on staff knowledge, attitudes, and practice: a cluster randomized trial J Am Geriatr Soc 2010 58 62 9 10.1111/j.1532-5415.2009.02629.x 20122041

[B20] Sandman PO Adolfsson R Norberg A Nyström L Winblad B Long-term care of the elderly. A descriptive study of 3600 institutionalized patients in the county of Vasterbotten, Sweden Compr Gerontol A 1988 2 120 132 3148369

[B21] Adolfsson R Gottfries CG Nyström L Winblad B Prevalence of dementia disorders in institutionalized Swedish old people. The work load imposed by caring for these patients Acta Psychiatr Scand 1981 63 225 244 6786003 10.1111/j.1600-0447.1981.tb00670.x

[B22] Folstein MF Folstein SE McHugh PR”Mini-mental state”: a practical method for grading the cognitive state of patients for the clinican J Psychiatr Res 1975 12 189 198 10.1016/0022-3956(75)90026-6 1202204

[B23] Lövheim H Sandman PO Karlsson S Gustafson Y Behavioral and psychological symptoms of dementia in relation to level of cognitive impairment Int Psychogeriatr 2008 20 777 89 18416871 10.1017/S1041610208006777

[B24] Eriksson L Pelling H Psykoser [In English: Psychoses] Läkemedelsboken. 2005/2006 2005 Stockholm: Apoteket AB 806 814

[B25] Cohen-Mansfield J Lipson S Werner P Billig N Taylor L Woosley R Withdrawal of haloperidol, thioridazine, and lorazepam in the nursing home: a controlled, double-blind study Arch Intern Med 1999 159 1733 1740 10.1001/archinte.159.15.1733 10448776

[B26] Ruths S Straand J Nygaard HA Aarsland D Stopping antipsychotic drug therapy in demented nursing home patients: a randomized, placebo-controlled study – The Bergen District Nursing Home Study (BEDNURS) Int J Geriatr Psychiatry 2008 23 889 895 10.1002/gps.1998 18306150

[B27] Brodaty H Ames D Snowdon J Woodward M Kirwan J Clarnette R Lee E Lyons B Grossman F A randomized placebo-controlled trial of risperidone for the treatment of aggression, agitation, and psychosis of dementia J Clin Psychiatry 2003 64 134 143 10.4088/JCP.v64n0205 12633121

[B28] Street JS Clark WS Gannon KS Cummings JL Bymaster FP Tamura RN Mitan SJ Kadam DL Sanger TM Feldman PD Tollefson GD Breier A The HGEU Study Group Olanzapine treatment of psychotic and behavioral symptoms in patients with Alzheimer disease in nursing care facilities: a double-blind, randomized, placebo-controlled trial Arch Gen Psychiatry 2000 57 968 76 10.1001/archpsyc.57.10.968 11015815

[B29] Gareri P Cotroneo A Lacava R Seminara G Marigliano N Loiacono A De Sarro G Comparison of the efficacy of new and conventional antipsychotic drugs in the treatment of behavioral and psychological symptoms of dementia (BPSD) Arch Gerontol Geriatr Suppl 2004 9 207 15 15207416 10.1016/j.archger.2004.04.029

[B30] Lövheim H Gustafson Y Karlsson S Sandman PO Comparison of behavioral and psychological symptoms of dementia and psychotropic drug treatments among old people in geriatric care in 2000 and 2007 Int Psychogeriatr 2011 23 1616 22 10.1017/S1041610211001712 21902862

[B31] Fossey J Ballard C Juszczak E James I Alder N Jacoby R Howard R Effect of enhanced psychosocial care on antipsychotic use in nursing home residents with severe dementia: cluster randomised trial Br Med J 2006 332 756 61 10.1136/bmj.38782.575868.7C 16543297 PMC1420717

[B32] O’Connor DW Ames D Gardner B King M Psychosocial treatments of psychological symptoms in dementia: a systematic review of reports meeting quality standards Int Psychogeriatr 2009 21 241 251 10.1017/S1041610208008223 19138459

[B33] Maidment ID Fox CG Boustani M Rodriguez J Brown RC Katona CL Efficacy of memantine on behavioral and psychological symptoms related to dementia: a systematic meta-analysis Ann Pharmacother 2008 42 32 38 18056833 10.1345/aph.1K372

[B34] Gauthier S Cummings J Ballard C Brodaty H Grossberg G Robert P Lyketsos C Management of behavioral problems in Alzheimer’s disease Int Psychogeriatr 2010 22 346 72 10.1017/S1041610209991505 20096151

[B35] Finkel SI Effects of rivastigmine on behavioral and psychological symptoms of dementia in Alzheimer’s disease Clin Ther 2004 26 980 990 10.1016/S0149-2918(04)90172-5 15336465

[B36] Pollock BG Mulsant BH Rosen J Sweet RA Mazumdar S Bharucha A Marin R Jacob NJ Huber KA Kastango KB Chew ML Comparison of citalopram, perphenazine, and placebo for the acute treatment of psychosis and behavioral disturbances in hospitalized, demented patients Am J Psychiatry 2002 159 460 465 10.1176/appi.ajp.159.3.460 11870012

[B37] Nyth AL Gottfries CG The clinical efficacy of citalopram in treatment of emotional disturbances in dementia disorders. A Nordic Multicentre study Br J Psychiatry 1990 157 894 901 10.1192/bjp.157.6.894 1705151

[B38] Lanctôt KL Herrmann N van Reekum R Eryavec G Naranjo CA Gender, aggression and serotonergic function are associated with response to sertraline for behavioral disturbances in Alzheimer‘s disease Int J Geriatr Psychiatry 2002 17 531 541 10.1002/gps.636 12112177

[B39] Pollock BG Mulsant BH Rosen J Mazumdar S Blakesley RE Houck PR Huber KA A double-blind comparison of Citalopram and Risperidone for the Treatment of Behavioral and Psychotic Symptoms Associated With Dementia Am J Geriatr Psychiatry 2007 15 942 52 10.1097/JGP.0b013e3180cc1ff5 17846102

[B40] Finkel S Kozma C Long S Greenspan A Mahmoud R Baser O Engelhart L Risperidone treatment in elderly patients with dementia: relative risk of cerebrovascular events versus other antipsychotics Int Psychogeriatr 2005 17 617 29 10.1017/S1041610205002280 16202186

